# Can complete blood count inflammatory parameters in epithelial ovarian cancer contribute to prognosis? - a survival analysis

**DOI:** 10.1186/s13048-019-0491-7

**Published:** 2019-02-11

**Authors:** Mehmet Ufuk Ceran, Umit Tasdemir, Eser Colak, Tayfun Güngör

**Affiliations:** 1 Department of Gynecology and Obstetrics, Baskent University School of Medicine, Konya Medical and Research Center, Selcuklu, Konya, Turkey; 2 Department of Gynecologic Oncology, Zekai Tahir Burak Women’s Health, Education and Research Hospital, Ankara, Turkey

**Keywords:** Epithelial ovarian cancer, Survival, PLR, NLR

## Abstract

**Subjective:**

The aim of the present study was to investigate the prognostic significance of preoperative complete blood count inflammatory markers in women operated for invasive Epithelial Ovarian Cancer (EOC).

**Method:**

Two hundred forty four patients that underwent operation with the diagnosis of invasive EOC between 2006 and 2014 were included in the study. The date of operation, date of recurrence and final mortality evaluations were performed for survival analysis. The sensitivity, specificity, PPV and NPV were separately calculated with ROC analysis. Survival analysis was carried out with Kaplan Meier-Log Rank Method.

**Results:**

Five-years overall survival rate was 56, 9% and 5-year disease-free survival (DFS) rate was 45,5%. Advanced disease stage, moderate-poor tumor differentiation, and the presence of recurrence were determined to have significant inverse relation at mean survival and 5-year survival rates. Neutrophil/lymphocyte ratio (NLR) and Platelet lymphocyte ratio (PLR) had prognostic effect on both DFS and overall survival based upon the cut-off values determined in the study (PLR = 231, s36, NLR = 3,83). Histopathological subtypes were not found to have any prognostic value. In correlation analysis, PLR and NLR had positive correlation with each other and negative correlation with overall survival.

**Conclusions:**

Inflammatory markers such as NLR and PLR have independent prognostic value for women who undergo surgery for invasive EOC.

## Introduction

Ovarian cancer is tenth most common one in all female cancers, and the fifth leading cause of cancer deaths in women. Moreover, it is the second most common type of gynecologic cancer and is the deadliest one [1]. Ovarian cancer has spread to sites has already metastasized outside the pelvis at the time of? diagnosis in 66% (two-third) of cases [2]. The majority (90%) of ovarian cancers are of epithelial cell origin [3]. The 5-year survival rate for patients with stage I and stage IV cancer disease? by qualified surgical staging are about 90%, 13,4%, respectively [4, 5]. The most important prognostic factors are the stage of disease at diagnosis and the residual tumor volume [2]. Pathogenesis is not very clear and some hypotheses have been proposed over the years. The incessant ovulation theory was first proposed by Fathalla in 1971 and emphasize the relationship between lifelong ovulation number and ovarian cancer [6]. Other hypotheses are the Gonadotropin Hypothesis based on high estrogen levels [7], the Inflammation Hypothesis [8], and the Retrograde Menstruation Theory [9]. The hypothesis on Type 1 and Type 2 molecular pathways in the development of epithelial cancer, which have been supported by these hypotheses, is accepted [10].

In recent years studies have focused on molecular basis of inflammation in cancer development. In recent cyto-genetic study, the working principle of immune system consisting of three phases (elimination, equilibrium and escape), working as dual host-protective and tumor-promoting actions, are emphasized. Tumor cells that defeat the immune response and pass through the 3rd phase are thought to originate from cancer stem cells (CSC) and are effective in resisting to treatment strategies [11]. Approximately two decades ago ıt was discussed that chronic inflammatory processes can induce carcinogenic pathway via oxidative damage on DNA that has mutagenic effect (such as p53 gene mutation) [12]. The more recent approach, however, is that a microenvironment and subsequent remodeling to transform epithelial cells through proinflammatory cytokines released after ovulation is involved in the onset and progression of Epithelial ovarian cancer (EOC) [13, 14]. In this process, oncogenic activation expressed by inflammation and proinflamatory transcription factors (such as NF-κB, STAT3 or HIF1α) cause inflamattory response network. Cytokines, chemokines (such as TNF-α, IL-6) and inflammatory enzymes (COX-2, cyclo-oxygenase 2) catalyze prostaglandin synthesis to function tumorogenesis process [15].

Our study is based on the Inflammation Hypothesis, which is believed to be the underlying mechanism for ovarian carcinogenesis [8]. Several studies have shown that elevated neutrophil count [16], platelet count [17], NLR [18, 19], and PLR [20] are associated with adverse clinicopathologic outcomes for many types of cancer including EOC. It is denoted that high levels of C-reactive protein may also be associated with ovarian cancer. [21]. So we considered to investigate the prognostic effect of an easily accessible and inexpensive inflammation marker such as neutrophil lymphocyte ratio (NLR) and platelet lymphocyte ratio (PLR). The aim of this study is to investigate whether preoperative complete blood count (CBC) inflammatory markers (PLR, NLR) have a prognostic value based on overall survival and disease-free survival (DFS) rates in patients who were histopathologically diagnosed with invasive EOC.

## Material and methods

This retrospective cohort study included the patients who underwent surgery in the Gynecological Oncology Clinic of Zekai Tahir Burak Women’s Health Research and Education Hospital between January 2006–December 2013 and who had an EOC based on histopathological examination. After approval of the study by Institutional Board of the hospital data was collected from hospital database. Patients’ age, parity, menopausal status, type and date of operation, chemotherapy treatment, date of recurrence (if there were recurrence symptoms) and survi status since February 2014 before the statistical analysis, histopathological subtypes, and stage and grade of the disease were recorded. CBCs and tumor markers that were taken prior to the operation or that were the closest to this date were also recorded. The WBC, neutrophil, lymphocyte, and platelet values were recorded separately from CBC results. Then, the neutrophil/lymphocyte and platelet/lymphocyte ratios to be studied were individually calculated.

Patients with clinical infectious diseases who had a high WBC count were excluded from the study. Patients with systemic diseases affecting WBC and platelet counts such as immunodeficiency and splenectomy were also excluded from the study.

The pathology results of the patients were examined. Accordingly, the patients who were diagnosed with invasive EOC were included in the study. Patients diagnosed with borderline epithelial tumors, sex cord-stromal tumors, germ cell tumors, and metastatic ovarian cancer were excluded from the study by considering their biological behaviors. In addition to the histological diagnosis of the patients, the stage of the disease and the degree of differentiation of the tumor were recorded.

During the follow-up after surgical cytoreduction, the criteria for presence of recurrence were determined based on imaging methods (ultrasound, computed tomography, magnetic resonance imaging), CA125 values, secondary cytoreduction, or results and findings of second-look laparotomy. Surgical staging for the patients was performed according to the 1988 FIGO staging system due to the retrospective nature of the study [22]. After surgical staging, Stage I and II disease were categorized as Early Stage, and Stage III and IV disease as Advanced Stage.

Final status assessment before survival analysis was performed based on the data of the Central Population Registration System within the Ministry of Interior General Directorate of Population and Citizenship Affairs if patient ID number was identified. It was performed by reaching the patients or their relatives via telephone if patient ID number could not be confirmed. DFS was defined as survival analysis considering the presence of recurrence symptoms in the patients. Overall survival was defined by considering patients survival status.

For statistical analysis The Statistical Package for the Social Sciences software (SPSS, version 21.0) was used. The Kolmogorov-Smirnov test, the Shapiro-Wilk test, and the coefficient of variability were used to examine whether the data showed a normal distribution. The Independent-samples t-test and the Mann-Whitney U (Exact) test were used to compare two independent groups. The One-Way Anova (Robust Test: Brown-Forsythe) was used to compare multiple groups with each other. The LSD test was used for post-hoc analysis. The Spearman’s RHO test was used to examine the correlations between the variables. The Pearson’s Chi-Square (Exact) test was used to compare categorical data. The effects of risk factors on mortality and survival time were analyzed by the Kaplan-Meier (product limit method) Mantel-Cox method. The Cox Regression (Enter Method- Backward Stepwise (Wald)) model was used to measure the effects of prognostic variables on survival time according to the main factor. The relationship between the classification, which was determined by the cut-off value calculated according to the variables of the patient groups, and the actual classification was established using the sensitivity, specificity, positive predictive value, and negative predictive value. These values were analyzed by the ROC Curve Analysis and then were expressed. Quantitative data were expressed as mean ± SD (standard deviation) and median ± IQR (interquartile range). Categorical data were expressed as number (n) and percentages (%). Data were analyzed at a 95% Confidence Interval, and a *p* value of < 0.05 was considered statistically significant.

## Results

Three hundred sixteen patients with malignant ovarian tumors who underwent surgery in the Gynecological Oncology Clinic were detected for analysis primarily. Seventy two patients who are not proper for the study according to criteria were excluded. 244 patients were included for final analysis.

**Fig. 1:**
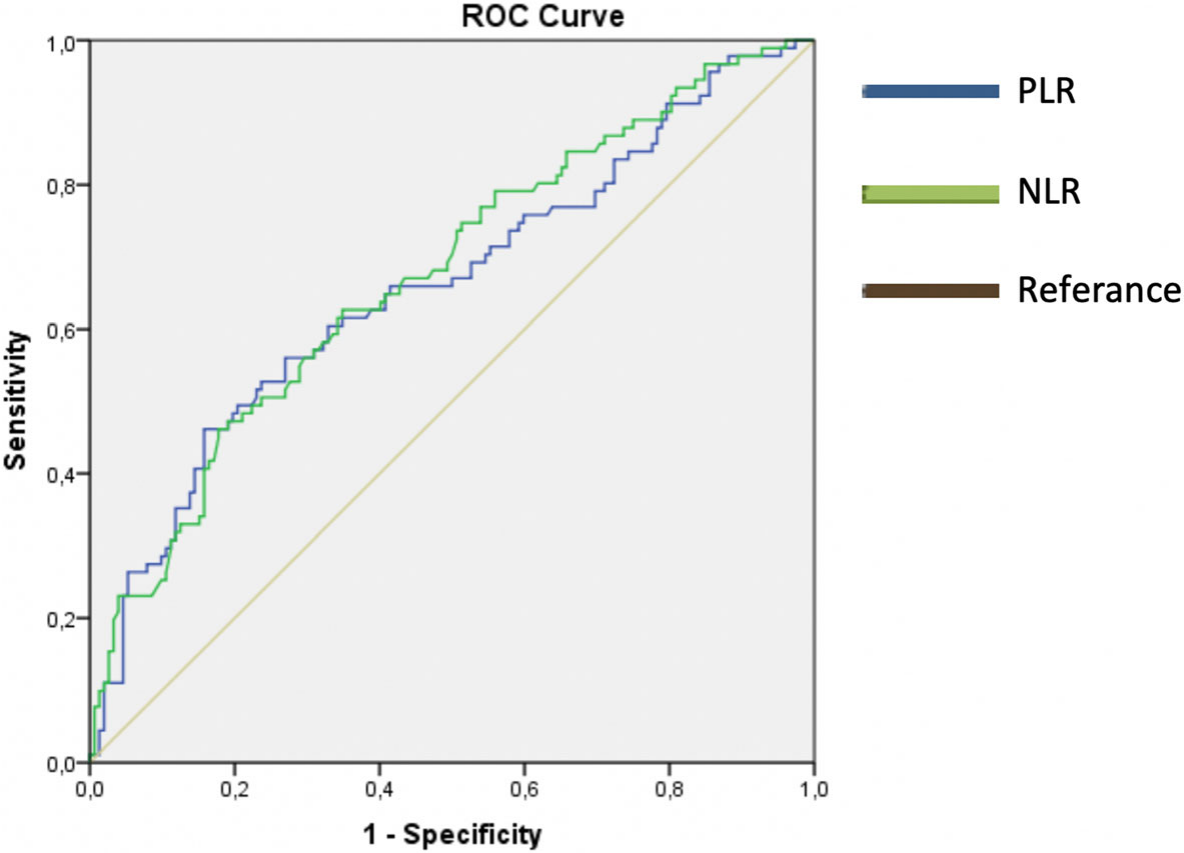
ROC curve for PLR and NLR according to cut-off values in terms of overall survival

The mean age of the patients was 52.4 ± 11 years (range: 26–86). Approximately 56% of the patients were diagnosed in the postmenopausal period. According to staging, 35.7% were diagnosed at Early Stage, and 64.3% at Advanced Stage (Table 1). The median values of PLR and NLR were 166.7 and 2.8, respectively. Median overall survival was calculated as 39.5 months, and the median DFS was calculated as 24.5 months. The cut-off values of PLR and NLR were 231 and 3.83, respectively (Table 2) PLR had the highest specificity with 84.2% (Fig. 1).

**Table 1:** Categorical characteristics of the patients in the study group

		Number (n:244)	Percent (%)
Age		mean ± sd	
Menopause	Premenopausal	106	43,4
	Postmenopausal	138	56,6
Stage	Early	87	35,7
	Ia	36	14,8
	Ib	4	1,6
	Ic	27	11,1
	IIa	13	5,3
	IIb	4	1,6
	IIc	3	1,2
	Advanced	157	64,3
	IIIa	3	1,2
	IIIb	3	1,2
	IIIc	104	42,6
	IV	47	19,3

**Table 2:** The cut-off, PPD, NPD, sensitivity, specificity values calculated according to the survival status of the patients in the study group in terms of the examined parameters

Variable	Cut Off value	Survival		*P* value
		Still alive	Exitus	
		n	n	
PLR	< 231,36	128	49	< 0,001
	> 231,36	24	42	
	Sensitivity/Specificity	PPV/NPV	AUC ± Se	
	46,2% / 84,2%	63,6%/72,3%	0,659 ± 0,037	
NLR	< 3,83	122	48	< 0,001
	> 3,83	30	43	
	Sensitivity/Specificity	PPV/NPV	AUC ± Se	
	47,3%/80,3%	58,9%/71,8%	0,674 ± 0,036	

Roc Curve Analysis, *Se* Standard error, *AUC* Area under the ROC curve, *PPV* Positive predictive value, *NPV* Negative predictive value

Based on PLR cut-off value; there was a significant difference for overall survival (median, IQR: 39.0 ± 37.0, 24.5 ± 32.0 months, *p* < 0.001) and DFS (median, IQR: 29.0 ± 35.0, 17.0 ± 19.0 months, *p* < 0.001),but no significant difference was observed for WBC count and hemoglobin value. The cut-off value of NLR was 3.83. Accordingly, the mean overall survival time was found to be 39.5 ± 37.0 months and 28.0 ± 32.0 months, respectively. Moreover, these values for DFSwere found to be 27.0 ± 35.0 months and 19.0 ± 23.0 months, respectively. The differences for both parameters were statistically significant.

Overall survival rate reduced significantly in patients with advanced stage disease, moderate or poor histopathological differentiation, platelet count more than 400,000/mm^3^, and over PLR-NLR cut-off values (Table 3). Advanced-stage disease, presence of recurrence, and over PLR cut-off value were found to increase mortality 7.66 times, 3.6 times, and 2.53 times, respectively. It was also concluded that they reduced overall survival rates. Similarly, a 1.19-fold increase was detected in mortality in the patients with thrombocytosis (platelet count> 400,000/mm^3^)(Table 4).

**Table 3:** The relationship between one, three and five year overall survival of patients and categorized parameters

Variable		Number of deaths	One year overall survival	Three years overall survival	Five years overall survival	*P* value
Stage	Early	8	98,90%	92%	90,10%	< 0,001
	Advanced	83	84,70%	58,40%	35,40%	
Grade	Well (1)	10	95,80%	86,70%	84,60%	P (1–2) < 0,001
	Moderate (2)	40	90%	68,80%	46,30%	P (2–3) =0,354
	Poor (3)	41	82,90%	57,90%	38,90%	P (1–3) < 0,001
PLT	≤400	54	92,30%	79,40%	64,90%	< 0,001
	> 400	37	80,60%	44,40%	33,20%	
PLR	≤231	49	92,10%	80,30%	66,00%	< 0,001
	> 231	42	81,80%	45%	34,20%	
NLR	≤3,83	48	92,90%	80%	66,30%	< 0,001
	> 3,83	43	80,80%	49,30%	36,70%	
General		91	26–89,3%	66–70,5%	84–56,9%	–

Kaplan Meier-Log Rank (Mantel-Cox), *PLT* Platelet count, *PLR* Platelet lymphocyte ratio, *NLR* Neutrophil lymphocyte ratio

**Table 4:** The factors affecting the overall survival of patients after multiple regression analysis

	B ± Se.	Odss Ratio [95% CI]	*P* value
Recurrence (Yes)	1281 ± 0,36	3,60 [1,78-7,28]	< 0,001
Stage (Advanced)	-2036 ± 0,60	7,66 [2,35-24,92]	0,001
Ca125 (> 307,25)	0,663 ± 0,26	1,94 [1152-3267]	0,013
Thrombocytosis (Plt > 400.000)	0,651 ± 0,24	1,19 [1,19-3,08]	0,007
PLR (> 231)	-0,928 ± 0,35	2,53 [1,28-4,99]	0,008

Cox Regression-Enter Method B: Regression Coefficient, *Se* Standard error, *CI* Confidence interval, *Plt* Platellet

Over PLR and NLR cut-off values, Advanced-stage disease, moderate and poor histopathological differentiation, thrombocytosis, and elevated CA125 level produced significant changes on disease-free survival. (Table 5). In multiple regression analysis, advanced-stage disease led to a 7.46-fold increase compared to early-stage disease, moderate and poor histopathological differentiation led to a 2.48-fold increase compared to well histopathological differentiation, and presence of thrombocytosis led to a 1.8-fold increase compared to absence of thrombocytosis, significantly. Correlation analysis showed that PLR was weakly and negatively correlated with overall survival and disease-free survival, and was moderately and positively correlated with CA125 value. There was no correlation between PLR and WBC count. Similar to PLR, NLR was weakly and negatively correlated with overall survival and disease-free survival, and was moderately and positively correlated with CA125 value. Unlike PLR, there was a moderate correlation between NLR and WBC count. A strong correlation was found between PLR and NLR.

**Table 5:** The relationship between one, three and five year disease-free survival of patients and categorized parameters

Variable		Number of Recurrence	One year disease free survival	Three years disease free survival	Five years disease free survival	Mean disease free survival	*P* Value
						Mean (month) ± Se	
Stage	Early	10	95,30%	91,30%	85,70%	85,076 ± 2,94	< 0,001
	Advanced	98	78,30%	32,50%	18,80%	33,167 ± 2,79	
Grade	Well (1)	12	94,20%	85,70%	78,90%	80,32 ± 3,82	P (1–2) < 0,001
	Moderate (2)	49	79,20%	47,40%	30,00%	42,25 ± 3,84	P (2–3) =0,384
	Poor (3)	47	82,40%	35,70%	28,50%	38,86 ± 4,18	P (1–3) < 0,001
PLT	≤400	71	89,30%	62,60%	51,60%	59,89 ± 3,09	< 0,001
	> 400	37	70,40%	30,40%	24,30%	33,54 ± 4,33	
PLR	≤231	66	88,60%	64,40%	55,20%	61,42 ± 3,13	< 0,001
	> 231	42	73,20%	28,80%	18,70%	33,75 ± 4,38	
NLR	≤3,83	65	89,30%	62,70%	54,50%	60,4 ± 3,22	< 0,001
	> 3,83	43	73,40%	37,30%	27,60%	39,1 ± 4,52	
General		108	34–84,8%	93–55,3%	106–45,5%	54,21 ± 2,73	–

Kaplan Meier-Log Rank (Mantel-Cox), *PLT* Platelet count, *PLR* Platelet lymphocyte ratio, *NLR* Neutrophil lymphocyte ratio

## Discussion

EOC is still the deadliest gynecological cancer due to the inability to fully understand the underlying biological mechanism. The overall 5-year survival rate varies between 31 and 53%. Although it depends on biological behavior of the tumor, factors associated with the patient, and the treatment applied, the prognosis is still not at the desired level [23, 24]. Chronic inflammation, defined as a risk factor in EOC, can occur in infections, autoimmune diseases, and benign and malignant tumors [25]. It is known that inflammation contributes to the development and progression of various cancers such as gastrointestinal system cancer [26], lung cancer [27], breast cancer [28], prostate cancer [29], especially pancreatic and colon cancer. In addition, DNA repair damage, relevant mutagens and many genetic studies are trying to elucidate the genetic map of ovarian cancer. Therefore, there are intensive studies on cancer immunoediting, molecular basis of inflammation, cytokines and expressed transcription factors [11, 13, 15]. The fact that inflammation is so up to date shows that easy and accessible inflammatory markers can be important in follow-up.

In a study conducted by Asher et al. on 235 patients undergoing surgery due to ovarian cancer, the cut-off values of PLR and NLR were determined as 300 and 4, respectively. They reported that elevated NLR and PLR were associated with poor prognosis in survival analysis [20]. In a multicenter study of Williams et al. (2014) involving 519 ovarian cancer patients, they reported that high NLR was associated with advanced-stage disease, moderate and poor histopathological differentiation, and poor prognosis. In the same study, they also noted that there was a high correlation between NLR and CA125 value [30]. In a study on this issue, Raungkaewmanee et al. examined 166 patients with invasive EOC who underwent surgery between 2004 and 2010. They emphasized that PLR was a better prognostic marker than both NLR and thrombocytosis, and that high PLR was associated with poor prognosis [31]. Cho et al. investigated the effect of NLR in predicting survival after treatment in ovarian cancer [18]. They suggested that NLR positivity (> 2.6 = cut-off) along with age and advanced disease stage were independent poor prognostic factors and that NLR positivity was the strongest predictor variable in the analysis [18]. Zhang et al. suggested that preoperative PLR was superior to other inflammatory markers such as CA 125, NLR, fibrinogen, C-reactive protein and albumin in ovarian cancer [32]. In a recent systematic review, it was concluded that PLR and NLR were promising but were not yet suitable for clinical use. They have proposed that the results should be reported consistently and comprehensively by excluding conditions affecting the immune system [33].

Cells responsible for inflammatory response, such as neutrophils, lymphocytes, and platelet, have been proposed as key factors in the recognition of tumorigenesis pathways [34]. We found PLR and NLR results similar to those reported in the literature. In our study, PLR and NLR had the highest specificity. CA125 had the highest sensitivity and the highest NPV (standard cut-off > 35). According to these results, we concluded that the standard value of CA125 was superior to the other parameters for screening purposes. Because of high rate of false positives, CA125 assessment by adding preoperative PLR and NLR parameters can help to make the correct diagnosis. Advanced-stage disease, moderate and poor histopathological differentiation, thrombocytosis, high PLR and NLR, and elevated CA125 level produced a significant difference on disease-free and overall survival, but histopathologic subtypes did not. The fact that PLR was not correlated with WBC count while NLR was moderately and positively correlated which can be considered as an advantage. PLR may be less affected by infections and autoimmune diseases that increase WBC count.

In conclusion, we can state that the elevation in PLR, NLR, and platelet count was significantly related with poor prognosis, advanced-stage disease, poor differentiation, and high-recurrence risk in survival analysis. Considering the infections that are frequently seen especially in the clinic and the factors that increase WBC count, the prognostic value of PLR must be appreciated. We think that PLR is a promising marker if large data such as the rate of maximal cytoreductive surgery, number of lifetime ovulation cycles, and platinum resistance are included in multivariate regression analysis and more patients are followed for a longer period.

## Abbreviations

DFS: Disease free survival
EOC: Epithelial Ovarian Cancer
NLR: Neutrophil lymphocyte ratio
NPV: Negative predictive value
PLR: Platelet lymphocyte ratio
PPV: Positive predictive value
WBC: White blood cell
